# 

**Published:** 1895-03-16

**Authors:** 


CONTENTS.

**Health by Act of Parliament** - 413
**The Definition of Puerperal Fever** - 414
**Aneurism.**—By A. PEARCE GOULD, F.R.C.S. - 415
**Social Problems.** I.—Society and the Beggar - 416
**On Modern Progress in Ophthalmic Medicine and Surgery.**—By R. B. CARTER, F.R.C.S. - 417
**The Plague of 1167** - 418
**Annotations.**—The Right Diet for John Bull—Old Age and Fatty Hearts—Influenza Ordinary and Extraordinary - 419
**Notes and News** - 420
**Scraps and Gleanings** - 430
**Medical Progress and Hospital Clinics:**
The Vaccination Question - 421
PROGRESS IN MEDICINE.—Infective Diseases - 423
Otology - 424
NEW APPLIANCES AND THINGS MEDICAL - 426
**The Institutional Workshop:**
HOSPITAL FINANCE - 427
HOSPITAL RE-CONSTRUCTION.—Devon and Exeter Hospital—Manchester Royal Infirmary - 427
HOSPITAL FESTIVALS AND MEETINGS - 428
**The Book World of Medicine and Science** - 431

EXTRA SUPPLEMENT.—THE NURSING MIRROR.

**News from the Nursing World.**—Royal Patients—Princess Christian at Oxford—Wake up! Eastbourne—Training at the London Hospital—Lewisham Infirmary—Royal National Pension Fund—East London Nursing Society—In Charge of an Inmate—Good Work in Sunderland—American Superintendents—A Martyr to Duty—Short Items - clxxi
**Elementary Anatomy and Surgery for Nurses.**—By W. MCADAM ECCLES, M.B., M.S., F.R.C.S. IX.—Special Fractures - clxxiii
**The Royal National Pension Fund for Nurses** - clxxiv
**Our American Letter** - clxxv
**Lewisham Infirmary** - clxxvi
**Royal British Nurses' Association** - clxvi
**Hints for Home Nursing** - clxvi
**Dress and Uniforms** - clxxvii
**Spring Novelties** - clxxviii
**The Nurse and the Sister** - clxxix
**Everybody's Opinion** - c.xxx

